# Case report: A case of effective treatment of primary myelofibrosis with nodular panniculitis using ruxolitinib combined with corticosteroids

**DOI:** 10.3389/fonc.2024.1412021

**Published:** 2024-08-19

**Authors:** Guzailinuer Wufuer, Jia-Lin Zhao, Qin Huang, Ainiwaer Babayi, Dilinuer Abudureyimu, Min Mao, Ming-hui Duan

**Affiliations:** ^1^ Department of Hematology, People’s Hospital of Xinjiang Uygur Autonomous Region, Urumqi, Xinjiang Uygur Autonomous Region, China; ^2^ Department of Pathology, People’s Hospital of Xinjiang Uygur Autonomous Region, Urumqi, Xinjiang Uygur Autonomous Region, China; ^3^ Department of Dermatovenereology, People’s Hospital of Xinjiang Uygur Autonomous Region, Urumqi, Xinjiang Uygur Autonomous Region, China; ^4^ Department of Hematology, Peking Union Medical College Hospital, Beijing, China

**Keywords:** subcutaneous nodule, primary myelofibrosis, nodular panniculitis, ruxolitinib, prednisone acetate

## Abstract

We report the case of a 54-year-old healthy Han Chinese male presenting with fever, pallor, erythematous subcutaneous nodules on the limbs, and significant anemia as indicated by routine blood tests, with no response to antimicrobial therapy. Initial skin biopsy was inconclusive. The erythematous subcutaneous nodules on the limbs rapidly progressed to widespread subcutaneous nodules across the body, with worsening anemia. Bone marrow biopsy revealed multifocal fibroblastic proliferation with focal fibrosis, classified as MF-2, and positive for the JAK2V617F mutation alongside SRSF2 positivity. Whole-body PET-CT scans did not reveal any lymph nodes or suspect lesions with high SUV uptake. A subsequent skin biopsy identified the condition as nodular panniculitis (NP), leading to a final diagnosis of primary myelofibrosis(PMF)with NP. The patient initially received treatment with oral ruxolitinib and prednisone acetate, resulting in normalization of body temperature, resolution of erythematous nodules, and normalization of blood parameters.

## Introduction

Nodular Panniculitis (NP) is a form of acute or subacute non-suppurative inflammation that originates within adipose tissue lobules (1). Predominantly affecting women, who constitute approximately 75% of cases, it can occur at any age but is most common between 30 to 50 years, with the incidence rate showing no racial disparities. The clinical manifestations of Nodular Panniculitis are diverse, leading to frequent misdiagnoses or underdiagnoses, and primarily include symptoms such as fever, fatigue, arthralgia, subcutaneous nodules or plaques, and may also impact multiple systems including respiratory, circulatory, and digestive. NP is often secondary to inflammatory, infectious, traumatic, and neoplastic diseases, although idiopathic cases, considered to be primary NP, do exist. Corticosteroids are recognized as the current effective treatment for this condition, yet their dosage and administration lack a standardized protocol, and the long-term efficacy remains uncertain. Only a minority of patients achieve long-term remission. The course of the disease in most patients is protracted and difficult to cure, with recurrent flare-ups (2). In some cases, severe visceral damage or secondary diseases can exacerbate the condition, potentially leading to mortality (3). PMF is a chronic myeloproliferative neoplasm characterized by progressive cytopenia, splenomegaly, systemic symptoms (such as fatigue, night sweats, fever), cachexia, bone pain, splenic infarction, pruritus, thrombosis, and hemorrhaging. Skin involvement in PMF is rare but may occasionally manifest as red plaques, nodules, erythema, ulcers, or bullae (4, 5). This report presents a case of NP concurrent with PMF and reviews the relevant literature on the co-occurrence of NP and PMF.

## Case report

### History

A 54-year-old male patient of Han ethnicity was admitted to the hospital on December 18, 2021, due to generalized skin rash accompanied by pain and intermittent fever lasting 15 days. Two weeks prior to admission, the patient developed erythematous rashes on his limbs and trunk without any apparent cause. These rashes were associated with swelling and continuous pain. Despite hospitalization and treatment at a local hospital, the patient’s condition did not improve. Upon admission, his hemoglobin was measured at 110 g/L, while leukocyte and platelet counts were within normal ranges. However, a progressive decline in hemoglobin levels was noted, along with the onset of fever, peaking at 40°C. Despite the anti-infective treatment of Cefoxin, the patient’s fever did not subside. Furthermore, topical application of traditional Chinese medicine to the rashes resulted in an increase in the number of lesions and a significant exacerbation of pain. The patient also experienced a weight loss of approximately 3 kg over one month.

**Figure 1:**
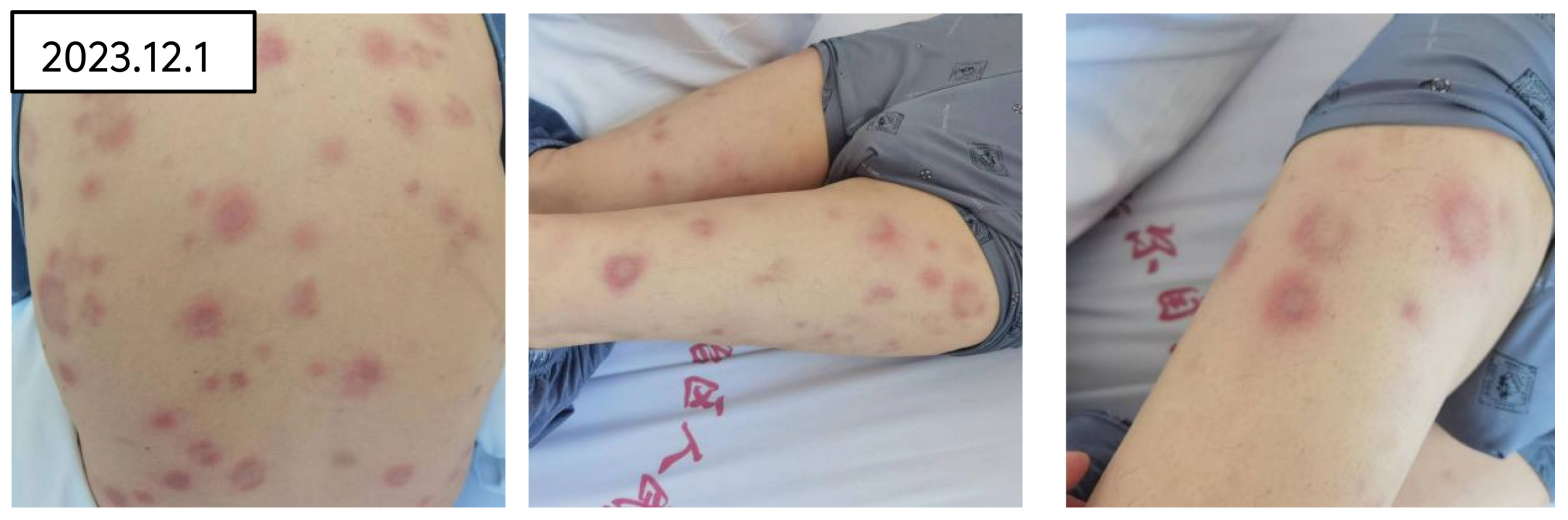
Skin rash of the patient on admission.

Upon admission, the physical examination revealed pallor suggestive of anemia, with sclerae not icteric. No petechiae were observed on the skin or mucous membranes. Scattered erythematous rashes of hard texture and tender on palpation were noted across the trunk and limbs (refer to **Figure 1**). On Systemic examination, CVS and Respiratory system were with in normal limits. The spleen was palpable 2cm below the costal margin, soft in texture, with a blunt edge and no tenderness on palpation. No hepatomegaly was detected, and there was no edema in the lower extremities.

Laboratory testing and imaging (**Table 1**):

**Table 1:** Laboratory testing and imaging.

**complete blood cell count (CBC):** white blood cell: 4.29×10^9^/L, hemoglobin: 73g/L, platelet 146×10^9^/L, mean corpuscular volume: 76.60 fL, neutrophil: 3.51×10^9^/L, lymphocyte: 0.61×10^9^/L. The blood film revealed immature granulocytes and elliptocytosis.
**C-reactive protein:** 77.14 mg/L.
**Comprehensive biochemical profiling:** Liver function tests were within normal limit, and lactate dehydrogenase: 629.31U/L.
**Coagulation function tests:** prothrombin time: 14.4s, activated partial thromboplastin time: 35.3s, fibrinogen levels: 4.27g/L, and D-dimer levels: 1.742mg/L DDU.
**Cytokine profiling:** interleukin-1β: 1.95pg/ml, interleukin-2: 1.15pg/ml, interleukin-4: 6.02pg/ml, interleukin-6: 28.52pg/ml, interleukin-10: 12.59pg/ml, interleukin-12p70: 2.79pg/ml, interleukin-17: 21.47pg/ml, interferon-γ: 8.03pg/ml, and tumor necrosis factor-α: 1.97pg/ml.
Screening for hepatitis A, B, and C, as well as HIV and RPR, yielded negative results. Multiple respiratory virus tests and two sets of blood cultures, as well as urine culture, were also negative.
**Additional tests:** erythropoietin levels: 356.64mIU/mL, vitamin B_12_: 349.00pg/mL, folate: 5.46ng/mL, and ferritin at 414.79ng/mL.
**Abdominal ultrasound:** splenomegaly of 15cm.

**Figure 2:**
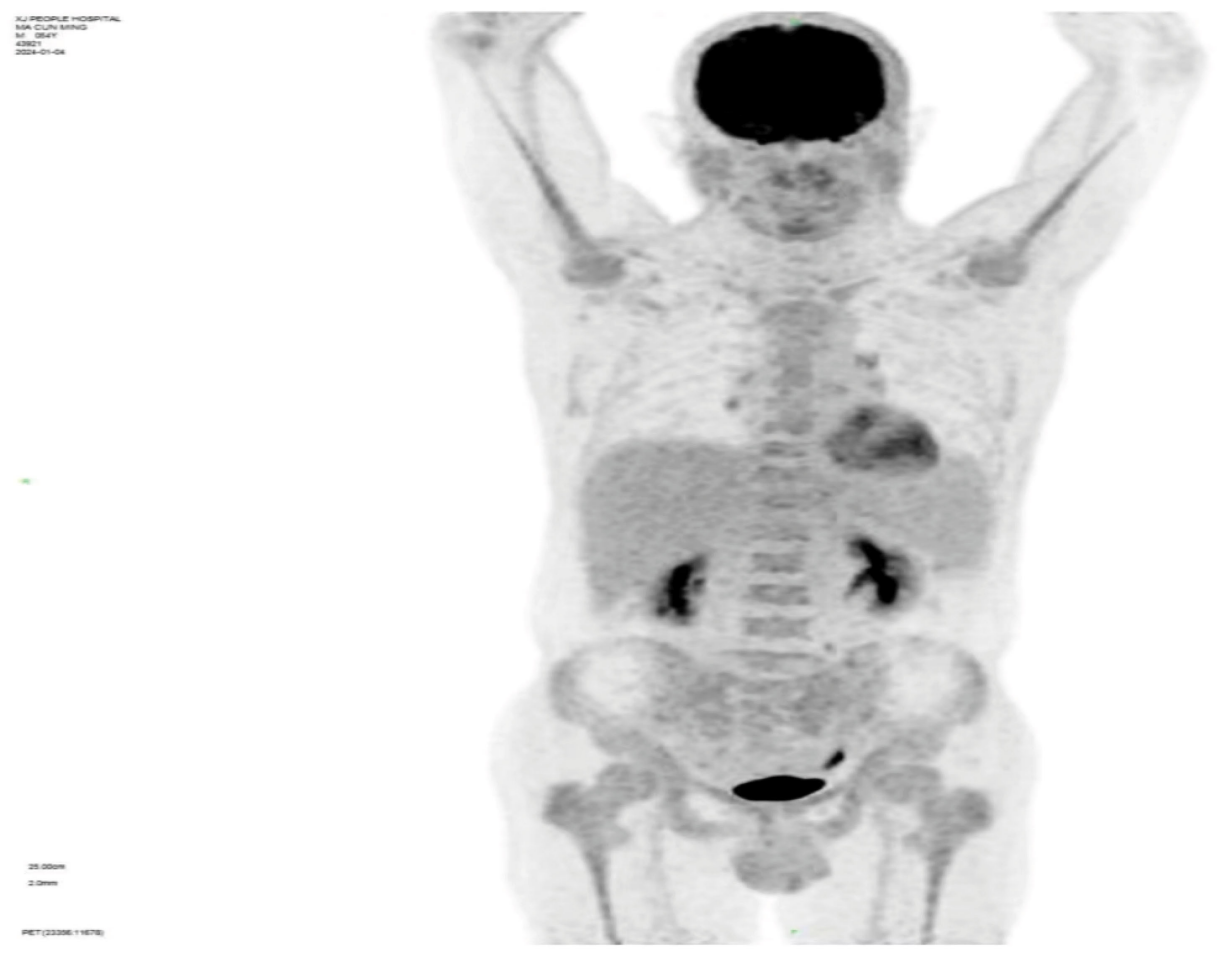
Whole-body PET-CT.

Whole-body PET-CT imaging reveals: 1) Diffuse FDG uptake elevation in both central and peripheral bone marrow, with uneven marrow cavity density observed in plain CT scans, indicating active bone marrow metabolism(SUV value4.6); 2) Splenomegaly without increased FDG metabolism; 3) Multiple small lymph nodes visible in the preauricular area of the left side, bilaterally below the jaw, the right clavicular region, axillae, and groin areas without elevated FDG metabolism (refer to **Figure 2**).

**Figure 3:**
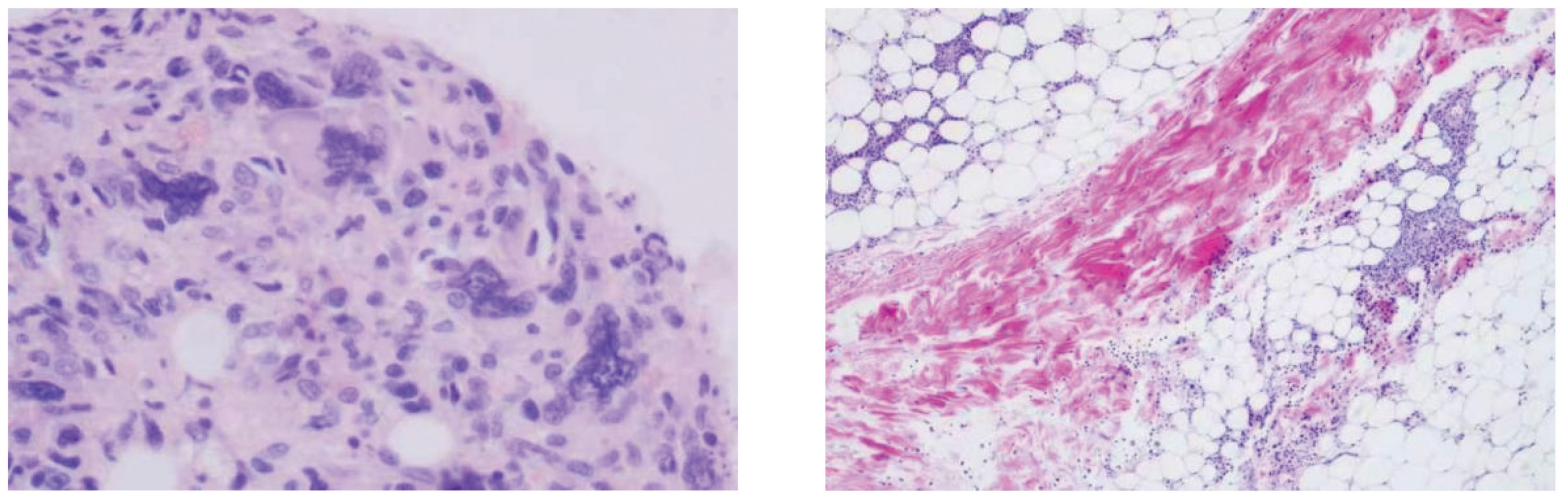
Bone marrow pathology: granuid erythroid hyperplasia, megakaryocyte hyperplasia (0–20/HPF), scattered or clustered, large and small megakaryocytes, online banking staining: grade 2.

**Figure 4:**
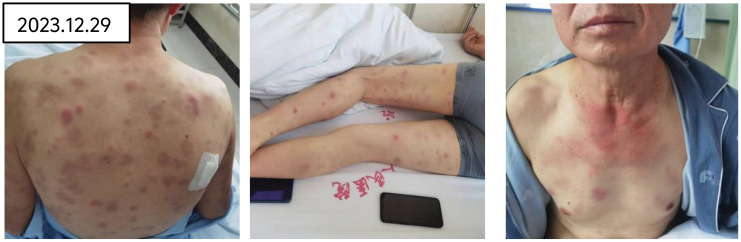
During the second skin biopsy 11 days after admission, the skin red nodules increased significantly and the pain worsened.

**Figure 5:**
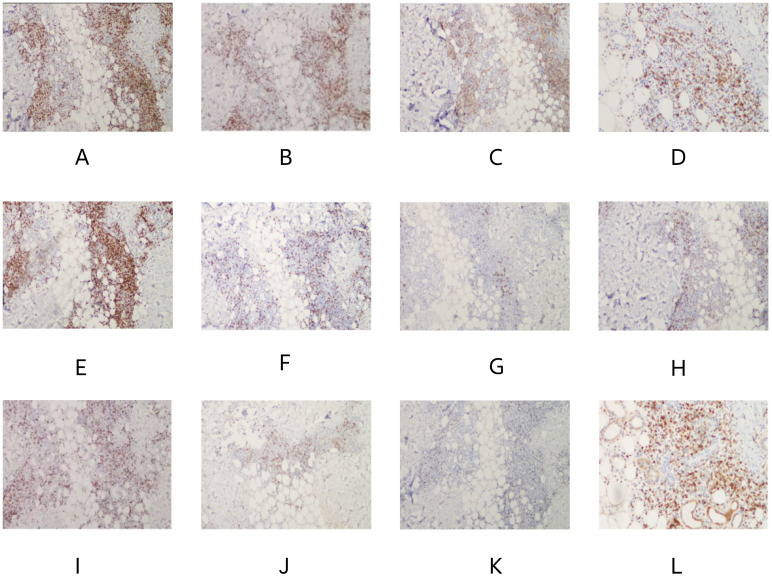
**(A)**, CD2 (+), **(B)**, CD3 (+), **(C)**, CD4 (+), **(D)**, CD5 (+), **(E)**, CD7 (+), **(F)**, CD8 (+), **(G)**, CD20 (minority +), **(H)**, CD56 (few +), **(I)**, Ki67 (20–30% +), **(J)**, GrB (+), **(K)**, TIA-1 (+), **(L)**, BCL2 (+). Result: (Right upper limb) Multifocal lymphocyte hyperplasia is seen subcutaneously, infiltrating between adipose tissue around the lamellar vessels, mixed with histiocytes. Combined with immunohistochemistry, it is considered as panniculitis.

Bone marrow smear analysis revealed: 1) Reduced marrow proliferation with a myeloid-to-erythroid ratio of 5:1. 2) Myeloid lineage constituted 68% with an increased proportion of mature granulocytes. 3) The erythroid lineage was reduced to 13.5%, predominantly consisting of intermediate to late erythroblasts, tear-drop and fragmented red cells observed. 4) Lymphocytes accounted for 15%, with no significant morphological abnormalities detected. 5) A total of four megakaryocytes were observed throughout the slide, with platelets being scattered and easily visible in small clusters. Bone Marrow Pathology: The hematopoietic tissue hyperplasia was significantly active, occupying approximately 90% of the volume. Adipose tissue hyperplasia decreased while myeloid system proliferation increased. However, red system proliferation decreased. Megakaryocyte hyperplasia (0–20/HPF) was observed, with scattered or clustered large and small megakaryocytes present, along with obvious fibrous tissue hyperplasia. Silver staining: Level 2 (refer to **Figure 3**) Conclusion: The diagnosis suggests PMF with bone marrow fibrosis (6). Chromosomal analysis yielded a karyotype of 46, XY[20]. Mutation analysis revealed JAK2 V617F positivity with a Variant Allele Frequency (VAF) of 64.47%. Comprehensive next-generation sequencing (NGS) for other myeloproliferative disorders was negative, culminating in a diagnosis of PMF with an IPSS score of 2, DIPSS score of 2, and MIPSS70 score of 3, classifying the patient as intermediate-2 risk.On December 21st, the initial biopsy of the left thigh skin revealed mild keratinization, slight spongiosis of the stratum spinosum, and the presence of scattered dyskeratotic cells within the epidermis. Moderate perivascular lymphocytic and rare eosinophilic infiltration was observed throughout the superficial and deep dermis extending to the subcutaneous tissue, with partial involvement of the vascular walls. Immunohistochemistry (IHC) showed positivity for CD3, rare positivity for CD20, a Ki-67 proliferation index of 5%, and rare CD138 positive cells. These findings, in conjunction with the patient’s clinical history, necessitate further follow-up.Given the discrepancy between the clinical presentation and the initial skin biopsy results, after thorough communication with the patient and their family, consent was obtained for a second skin biopsy. Conducted on December 29th on a rash located on the back, the pathology of the second biopsy revealed no dysplasia in the epidermal squamous layer (refer to **Figure 4**). The dermis showed infiltration by lymphocytes and mononuclear cells around small blood vessels and adnexal structures, with a minor presence of neutrophils. Similar infiltrates were observed around small vessels in the subcutaneous adipose tissue without evident liponecrosis. The lymphocytes appeared mature with irregular nuclei. Perineural infiltration by inflammatory cells, resembling panniculitis, was noted. Immunohistochemical analysis corroborated the diagnosis of panniculitis (refer to **Figure 5**).

**Figure 6:**
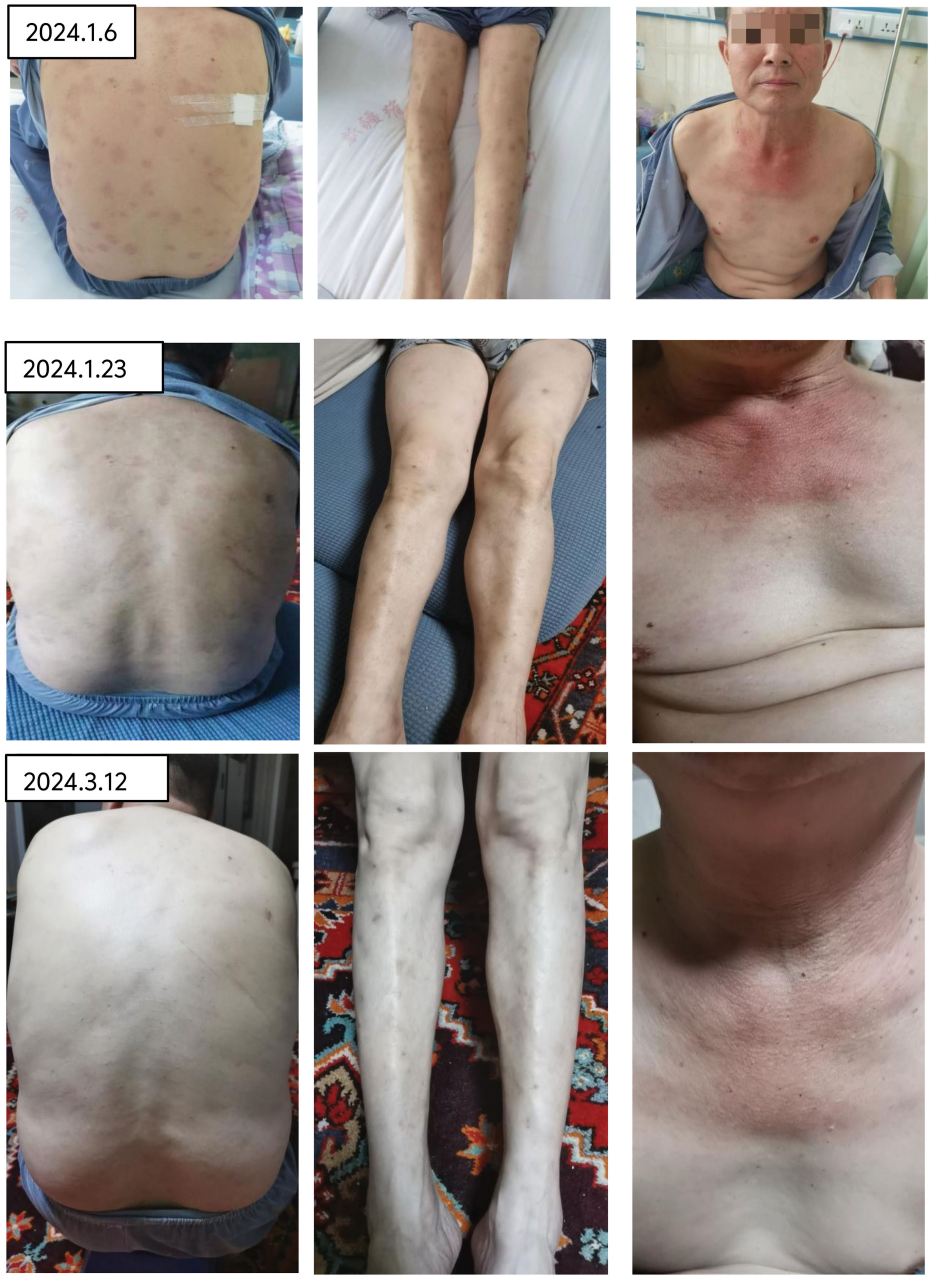
After treatment with rutinib and prednisone acetate, the rash gradually faded and the pain disappeared.

Therapeutic interventions initiated on January 2, 2024, including the administration of ruxolitinib at a dosage of 15 mg twice daily and prednisone at 65 mg per day, resulted in the normalization of the patient’s body temperature, a gradual fading of the rash, and the alleviation of pain (refer to **Figure 6**). Additionally, hemoglobin was observed to rise to 120g/l.

## Discussion

Primary Myelofibrosis (PMF) is a myeloproliferative neoplasm (MPN) characterized by the clonal proliferation of aberrant hematopoietic stem cells, leading to progressive bone marrow fibrosis. This condition is often associated with mutations in JAK2, CALR, or MPL, though not exclusively. PMF manifests through a spectrum of pathological features, including reticulin and/or collagen fibrosis in the bone marrow, extramedullary hematopoiesis (EMH), and more (7, 8). Extramedullary hematopoiesis can potentially occur in almost any organ system in PMF patients (9–11). Skin manifestations in PMF can include erythematous patches, nodules, erythema, ulcers, or bullae, often associated with extramedullary hematopoiesis, blast crisis, or Sweet’s syndrome. To our knowledge, there are no reports of PMF secondary to NP.

NP is characterized by erythema, plaques, or subcutaneous nodules, with some instances of panniculitis accompanied by pain or ulceration. It may also manifest with varying degrees of systemic symptoms, such as fever, joint pain, and fatigue. Diagnosis of NP necessitates a correlation between clinical presentation and histopathological examination from tissue biopsies (12). In cases with a high index of suspicion, multiple biopsies may be required. This case underscores the pivotal role of histopathological examination, as the patient was diagnosed and effectively treated following two skin biopsies. In a minority of cases, extensive organ involvement precedes cutaneous manifestations, with the digestive system being a common site of affliction among others (13). Etiologically, NP can be primary or secondary, with the latter often resulting from inflammation, infection, trauma, deposition diseases, enzymatic destruction, or malignancies (12, 14).

Recent studies suggest that NP, when co-occurring with autoimmune diseases such as SLE, myositis, SS, and vasculitis, acts as a secondary dermatological manifestation rather than a single disease (15). Instances of NP coinciding with myeloid hematological malignancies are sporadically reported, indicating a potential secondary relationship to these malignancies. For example,Alexa J. Cohen (16) reported a case of a 65-year-old female patient presenting with limb pain and pruritic nodules, who was eventually diagnosed with NP concomitant with MDS/MPN. After one month of oral corticosteroid therapy, the nodules completely resolved. Gao Huaqiang (17) reported a case of a 71-year-old female presenting with anemia, thrombocytopenia, and multiple lower limb rashes, who was ultimately diagnosed with NP associated with MDS EB-1. The rashes resolved after treatment with oral prednisone, but the patient progressed to acute leukemia five months later. Paolo Fraticelli and colleagues (18) reported a case of a 60-year-old male patient exhibiting fever, leukocytosis, and pain and swelling in the right calf with subcutaneous nodules. The final diagnosis from the skin biopsy was NP accompanied by atypical chronic myeloid leukemia. The condition was similarly treated with high-dose intravenous corticosteroid injections, resulting in significant improvement in skin lesions and normalization of blood tests. However, the diagnosis of this atypical CML case lacked genetic evidence, complicating differentiation from leukemoid reaction secondary to NP. The case we report not only exhibited grade 2 myelofibrosis but also harbored a JAK2 V617F mutation with a high VAF, thereby confirming a diagnosis of PMF. With two definitive diagnoses of NP excluding other possibilities such as Sweet’s syndrome, and sustained response to a treatment regimen primarily comprising hormones and ruxolitinib, this case can be conclusively identified as PMF secondary to NP.

Patients with secondary NP may require concurrent treatment for both the primary condition and NP. However, there is currently no specific treatment for NP itself. During acute inflammatory phases or in cases presenting with high fever, corticosteroids typically demonstrate significant efficacy. Beyond corticosteroids, non-steroidal anti-inflammatory drugs (NSAIDs) are effective in managing joint pain and the discomfort and fever associated with subcutaneous nodules. For systemic NP, particularly in severe cases, the concurrent use of one or two immunosuppressants is recommended, alongside appropriate management of visceral involvement and enhanced supportive care. Commonly used immunosuppressants include methotrexate, cyclophosphamide, hydroxychloroquine, leflunomide, azathioprine, cyclosporine, mycophenolate mofetil, and Tripterygium wilfordii polyglycosides. In the case presented, the patient’s body temperature normalized and the rash improved following treatment with corticosteroids in combination with ruxolitinib. While ruxolitinib is targeted for primary myelofibrosis (PMF) and its mechanism does not specifically target the JAK2 V617F mutation, it achieves significant splenic reduction and symptom relief by attenuating the activity of the JAK-STAT pathways (19). The reduction in JAK-STAT pathway activity leads to decreased levels of multiple cytokines, endowing ruxolitinib with a marked immunosuppressive effect. This has already been successfully utilized in treating conditions such as graft-versus-host disease and hemophagocytic lymphohistiocytosis. We hypothesize that the therapeutic efficacy of ruxolitinib in this case is primarily due to its immunosuppressive mechanism. It is noteworthy to consider whether ruxolitinib may serve as a second-line treatment option for other types of NP, warranting further investigation.

## Conclusion

In summary, the patient in this case presented with subcutaneous nodules and fever as initial symptoms and was ultimately diagnosed with NP following two skin biopsies. Despite the absence of proliferative changes in peripheral blood cells, the presence of grade 2 MF in the bone marrow, along with a high VAF of the JAK-2V617F mutation, was observed. Treatment with steroids in combination with ruxolitinib resulted in significant improvement, suggesting the presence of NP may be secondary to the diagnosis of PMF, marking the first report of such a case internationally. This case underscores the importance of identifying underlying etiologies in confirmed cases of NP to achieve optimal therapeutic outcomes through a comprehensive treatment approach. Moreover, the potential of ruxolitinib as a second-line treatment option for NP warrants further in-depth investigation.

## Data Availability

The original contributions presented in the study are included in the article/supplementary materials. Further inquiries can be directed to the corresponding authors.
